# *Corallodiscus flabellata* B.L. Burtt Extracts Stimulate Diuretic Activity and Regulate the Renal Expression of Aquaporins

**DOI:** 10.1155/2020/6020817

**Published:** 2020-02-27

**Authors:** Yuxuan Kan, Mengnan Zeng, Beibei Zhang, Benke Li, Shenchao Wang, Yangyang Wang, Ruiqi Xu, Yuanyuan Wu, Xiaoke Zheng, Weisheng Feng

**Affiliations:** ^1^Department of Medicine, Henan University of Chinese Medicine, Zhengzhou 450046, China; ^2^Co-construction Collaborative Innovation Center for Chinese Medicine and Respiratory Diseases, Henan & Education Ministry of China, Zhengzhou 450046, China

## Abstract

*Corallodiscus flabellata* B. L. Burtt is a traditional Chinese medicine. Previous studies in our laboratory showed that *C. flabellata* alleviated symptoms of Alzheimer's disease (AD) in a rat model of AD and increased healthy rats' urine volume. The aims of this study were to explore the diuretic activity of different extracts from *C. flabellata* and to identify the underlying mechanisms of action. Different doses of a *C. flabellata* extract (CF-L, CF-M, and CF-H) were administered orally to male KM mice in a single dose. In another procedure, *C. flabellata* (CF), water extract, and 20%, 30%, and 40% ethanol extracts of *C. flabellata* (CF-WE, CF-20, CF-30, and CF-40) were administered orally daily for 5 days. The urinary excretion rate, osmolality, and electrolyte levels in urine and serum, renal expression of aquaporins (AQPs), apoptosis-related protein, and MAPK-related protein were analyzed. The results showed that single doses of CF-M and CF-H increased urinary volume significantly, as well as daily administration of CF, CF-WE, CF-20, CF-30, and CF-40. Furthermore, CF-20 and CF-30 increased the concentration of Na^+^ in the urine. Treatment with CF-40 increased the urine osmolality and Na^+^ and Cl^−^ concentrations and decreased the concentration of Na^+^ in the serum. Also, CF, CF-WE, CF-20, CF-30, and CF-40 decreased the renal expression of AQPs, as well as the ratios of Bcl-2/Bax, p-ERK/ERK, p-JNK/JNK, and p-p38/p38. In sum, the medium and high doses of the *C. flabellata* extract and CF-WE, CF-20, CF-30, and CF-40 were found to have a diuretic activity. They may inhibit the renal expression of AQPs and apoptosis-related proteins by inhibiting the MAPK signaling pathway, thereby achieving diuretic effects.

## 1. Introduction

*Corallodiscus flabellata* B. L. Burtt, whole plants of *Corallodiscus flabellata*, also called Shihua and Shihudie, occurs mainly in Guangxi, Yunnan, Hubei, and Henan Provinces. Its extracts are known to support blood circulation and detoxification, relieve swelling and pain, and reduce dampness and heat. In Henan, the extracts are used for treating early upper respiratory tract infections during colds and irregular menstruation or abnormal leucorrhea in women [1]. In *Kunming folk medicinal herbs most in use*, the extracts are considered to eliminate heat and toxicity; reduce dampness, parotitis, and sore throat; and cure arthralgia syndrome due to dampness and heat. Several studies found that the *C. flabellata* contains C-glycosyl flavones [2], phenylethanoid glycosides [3], phenolic acids [4], and other compounds. The pharmacological actions of C-glycosyl flavones may have antioxidative, antiradiation, cardiovascular protective, and hypoglycemic effects [5]. Phenylethanoid glycosides exhibit a wide range of curative bioactivities, such as antibacterial, anti-inflammatory, antiviral, and neuroprotective, as well as other effects [6]. Previous studies in our laboratory showed that *C. flabellata* increased rats' urine volume and alleviated symptoms of Alzheimer's disease (AD) in a rat model of AD. However, thus far, no relevant report is available on the diuretic effect of *C. flabellata*. Therefore, in the present study, we assessed the diuretic activity of *C. flabellata* and the possible mechanisms underlying these effects.

Urinary system disease is most common in the elderly. Because China's demography is currently entering an era marked by aging, the overall demand for therapeutic drugs is expected to expand further. Consequently, research and development of new drugs for the treatment of urinary system diseases have significant social and economic benefits. Importantly, the currently used diuretic drugs such as furosemide and thiazide, which act on the kidney and increase electrolyte and urine output, may produce toxic side effects such as electrolyte imbalance and, possibly, irreversible functional organ damage after prolonged use [7]. The purpose of the present study was to explore the diuretic activity of different fractions extracted from *C. flabellata* and to examine the possible underlying mechanisms. These observations should provide theoretical guidance in the development of novel diuretics.

## 2. Materials and Methods

### 2.1. Plant Material and Extraction

The *Corallodiscus flabellata* B. L. Burtt were obtained from the Funiu Mountain area of Xixia County, Henan Province, and identified by Prof. Suiqing, Chen, Henan University of Chinese Medicine. A voucher specimen (No. 20171021A) was deposited in the Research Department of Natural Medicinal Chemistry, School of Pharmacy, Henan University of Chinese Medicine.

Whole plants of *C. flabellata* (1 kg) were mixed with 50% ethanol (at a 1 : 12 ratio), and the mixture was boiled three times under reflux for 1 h at 100°C. A 50% ethanol extract was obtained and dissolved in 80% ethanol, after which the insoluble matter was separated by centrifugation and discarded. The supernatant was fed through a Diaion HP-20 macroporous resin column followed by consecutive elutions with water, 20% ET, 30% ET, and 40% ET, which yielded different fractions. Then, these extracts were treated by freeze-drying process, and they contained no ethanol. Finally, the extraction rates of these fractions were 4.1%, 1.0%, 1.0%, and 1.8%. The extracts were diluted in distilled water to obtain appropriate concentrations for intragastric administration.

### 2.2. Reference Drug

Hydrochlorothiazide, a medium-acting diuretic, was used as the positive control drug (Chang Zhou Pharmaceutical Co., Ltd., China). It was dissolved in water to a concentration of 4 mg/ml prior to administration.

### 2.3. Experimental Animals

Male KM mice (18–22 g) were obtained from Beijing Vital River Laboratory Animal Technology Co., Ltd. All the animals were housed under standard conditions (18–22°C, humidity 55 ± 5%, and a 12 h/12 h light/dark cycle). The animals had ad libitum access to water and standard mouse food. The experiments were conducted in accordance with Experimental Animal Administration regulations issued by the State Committee of Science and Technology of the People's Republic of China (ethical approval reference number: SYXK2015-0005). Mice were placed individually in metabolic cages for adaptive feeding for two days preceding the experiments. They were allowed to drink freely but given no access to food for 18 h. Gentle manual pressure on the abdomen was exerted to remove residual urine from the bladder. Before the treatment, all animals received physiological saline at a dose of 0.2 mL/10 g body weight by oral gavage to obtain uniform water and salt load and to facilitate the collection of significant volumes of urine 2 h after treatment. Mice whose urine volume was more than 40% saline volume were included in the further experiment [8].

### 2.4. Acute Diuretic Activity

Metabolic cage methodology was used to study diuretic activity [9]. For examination of the acute diuretic activity, mice were divided into five groups (*n* = 8), matched for body weight and urine volume: the control group (Con), hydrochlorothiazide group (40 mg/kg, HCTZ), and low-, medium-, and high-dose *C. flabellata* groups (77.5 mg/kg, CF-L; 155 mg/kg, CF-M; and 310 mg/kg, CF-H, respectively). Thirty minutes before drug administration, all mice were administered 0.2 mL/10 g body weight (BW) of isotonic saline to impose uniform water and salt load by oral gavage. Thirty minutes later, drugs were administered to the different experimental groups of mice by oral gavage, whereas control animals received the same volume of distilled water. The urine was collected in graduated cylinders, and the sample volume was recorded 1, 3, 5, 7, 9, and 11 h after administration of the drug. The recorded volumes were transformed into ratios of cumulative urinary excretion to body weight, expressed as mL/25 g, for further analysis of between-group differences.

### 2.5. Subchronic Diuretic Activity

The mice used in the experiment examining subchronic diuretic activity were allocated to seven groups (*n* = 8), which were matched for body weight and urine volume. These groups received either *C. flabellata* (310 mg/kg, CF), water extract of *C. flabellata* (82 mg/kg, CF-WE), or 20%, 30%, or 40% ethanol extract of *C. flabellata* (20 mg/kg, CF-20; 20 mg/kg, CF-30; and 36 mg/kg, CF-40). Drug administration was performed daily for five consecutive days by oral gavage, as described above. On each day, urine volume was collected for 7 h following drug administration.

#### 2.5.1. Sample Collection

The collected urine samples were centrifuged (1000 × g at 4°C) and stored at −80°C until analysis. Blood samples were collected by retro-orbital bleeding. Serum was obtained by centrifugation (3000 rpm for 10 min at 4°C) and stored at −20°C until analysis. Concentrations of sodium (Na^+^), potassium (K^+^), and chlorine (Cl^−^) in urine and serum were analyzed by flame spectrophotometry. The osmolalities of urine and plasma were determined by an Osmometer (OM815; Loser Messtechnik).

#### 2.5.2. Western Blotting

The animal kidneys were removed on both sides, and the serosal surface layer was carefully peeled off. Then, proteins were isolated from the kidneys using a total protein extraction kit (Beijing Solarbio Science & Technology Co., Ltd., China). Next, proteins were quantified using the BCA protein assay kit (Beijing Solarbio Science & Technology Co., Ltd., China). Samples were separated on 4% and 12% SDS gels and transferred onto nitrocellulose membranes. The membrane was sealed on a shaker with 5% skim milk (BD Co., UK) in PBST (PBS containing 0.1% Tween 20) for 1 h and then incubated with the appropriate primary antibodies for 2 h at room temperature, the primary antibodies being AQP1 (1 : 500, Abcam, MA, USA), AQP2 (1 : 500, Abcam, MA, USA), AQP3 (1 : 500, Abcam, MA, USA) AQP4 (1 : 500, Abcam, MA, USA), ERK (1 : 1000, Abcam, MA, USA), p-ERK (1 : 1000, Abcam, MA, USA), JNK (1 : 2000, Abcam, MA, USA), p-JNK (1 : 5000, Abcam, MA, USA), p38 (1 : 1000, Abcam, MA, USA), p-p38 (1 : 1000, Abcam, MA, USA), Bax (1 : 10,000, Abcam, MA, USA), and Bcl-2 (1 : 1000, Abcam, MA, USA). After washing five times with PBST, membranes were incubated with Alexa Fluor®-linked secondary antibodies (1 : 2000 dilution with PBST containing 5% skim milk) at room temperature for 1-2 h. The protein bands were collected and analyzed with Odyssey® CLx Near-Infrared Imaging System (Li-COR Biosciences Co., UK). Next, the membrane is submerged in appropriate amount of 1x Antibody Stripping Solution (Cat.No. 2500, Merck Millipore, USA) and incubated with gentle mixing for 15 minutes at room temperature. Then, the membrane was sealed on a shaker with 5% skim milk in PBST. After washing two times each with PBST, the blot is ready for reprobing with another antibody.

#### 2.5.3. Immunofluorescence

The renal tissues were fixed with 4% paraformaldehyde made in PBS (pH 7.2), embedded in paraffin, and cutted into 5 *μ*m sections. After 1 h blocking with appropriate 10% (v/v) normal serum at room temperature, the slides were incubated with primary antibodies of AQP1, AQP2, AQP3, and AQP4 (as above) at ratio of 1 : 100 overnight at 4°C. After washing three times, slides were incubated with fluorophore-conjugated secondary antibodies (LI-COR Biosciences Co., UK) at a ratio of 1 : 300 for 1 h at room temperature. The wavelength of the Licor antibody is 700 nm. Fluorescence signals were visualized and captured by using Cytation 5.

### 2.6. Statistical Analysis

The results were expressed as the mean ± SD (standard deviation). Statistical analysis was performed using SPSS 20.0 software (IBM, Armonk, NY). Differences between the groups receiving control and experimental treatments were assessed by analysis of variance (ANOVA), followed by Dunnett's *t*-test for multiple comparisons. *P* values smaller than 0.05 were considered to be statistically significant.

## 3. Results

### 3.1. Urinary Volume of Acute Diuretic Experiment

*C. flabellata* extracts were assessed for their diuretic effects after acute oral administration in the saline-preloaded mice (Table 1).

The results show that CF-M and CF-H increased urinary volume significantly (*P* < 0.05 and *P* < 0.01, respectively). The diuretic activity of CF-M and CF-H was seen primarily 5 h after administration, while the diuretic activity of HCTZ occurred mainly during the first 7 h.

### 3.2. Urinary Volume of Subchronic Diuretic Experiment

The effects of different extracts of *C. flabellata* were also examined following continuous administration in the saline-loaded mice (Table 2). Compared with the control group, animals receiving CF-WE only increased the urinary volume on the first day of treatment (*P* < 0.05), whereas animals receiving CF, CF-20, and CF-30 increased urinary volume on the second day (*P* < 0.05, *P* < 0.01). These results indicate that the extracts may have rapid and short-lasting diuretic activity. Throughout the five-day treatment period, a statistically significant diuretic effect of CF-40 was observed (*P* < 0.05, *P* < 0.01). No significant differences in diuretic effects were observed between animals treated with CF-40 and those treated with HCTZ. HCTZ is a classical diuretic agent. This implies that CF-40 has long-lasting diuretic activity.

### 3.3. Effects on Osmolality of Urine and Serum

Table 3 shows the osmolalities of urine and serum after oral administration of different fractions extracted from *C. flabellata*. Compared with the control group, the groups treated with CF-40 and hydrochlorothiazide increased the urinary osmolality significantly (*P* < 0.05). However, treatment differences in effects on the serum osmolality did not reach statistical significance.

### 3.4. Effects on Electrolyte Levels in Urine and Serum

The effect of different fractions extracted from *C. flabellata* on urinary electrolyte (Na^+^, K^+^, and Cl^−^) levels is summarized in Table 4. Compared with the control group, mice receiving CF-20 and CF-30 showed significantly increased urinary Na^+^ concentrations (*P* < 0.05). CF-40 increased the concentration of both Na^+^ and Cl^−^ significantly (*P* < 0.05), and its effects were similar to those of HCTZ. The urinary concentration of K^+^ did not show significant changes in response to the various treatments.

Effects of different fractions extracted from *C. flabellata* on serum electrolyte (Na^+^, K^+^, and Cl^−^) concentrations are shown in Table 5. Serum concentrations of Na^+^ were reduced in mice treated with CF-40 and HCTZ, relative to mice in the control group (*P* < 0.05). However, the concentration effects of K^+^ and Cl^−^ did not reach statistical significance.

### 3.5. Effects on the Expression of AQPs in the Kidney

**Figure 1:**
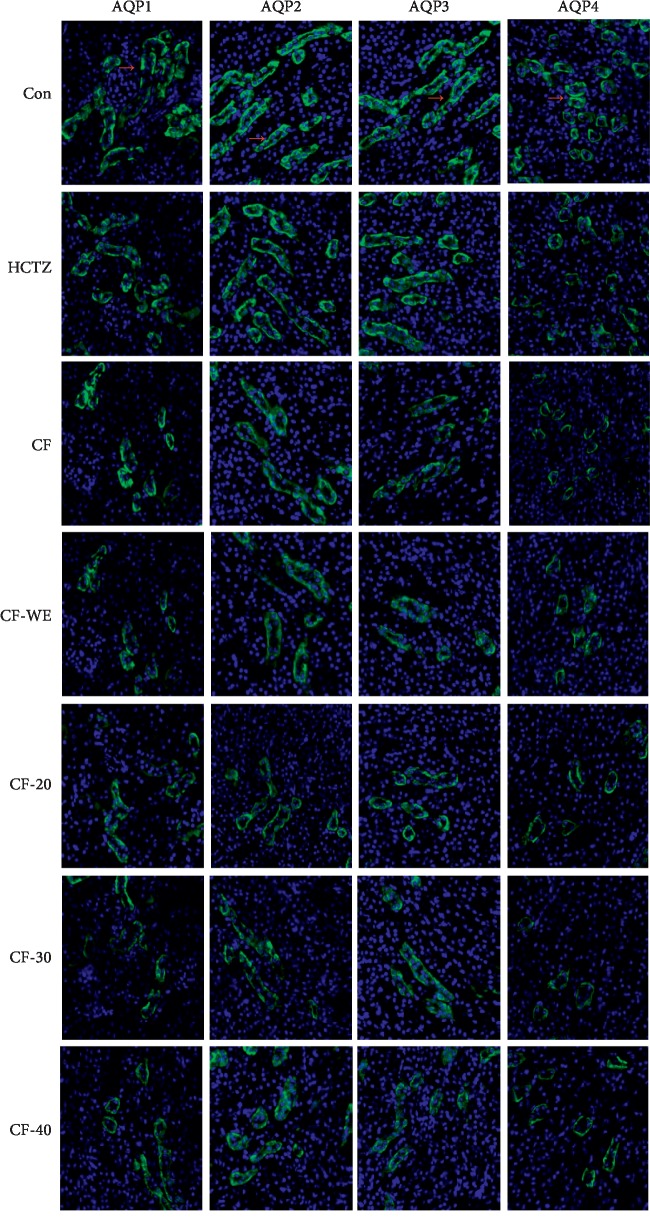
The distribution of AQPs in the kidney as shown by immunofluorescence staining (40x). First column: AQP1 (green). Second column: AQP2 (green). Third column: AQP3 (green). Fourth column: AQP4 (green). Red arrowheads show the protein distribution (*n* = 3 per group).

**Figure 2:**
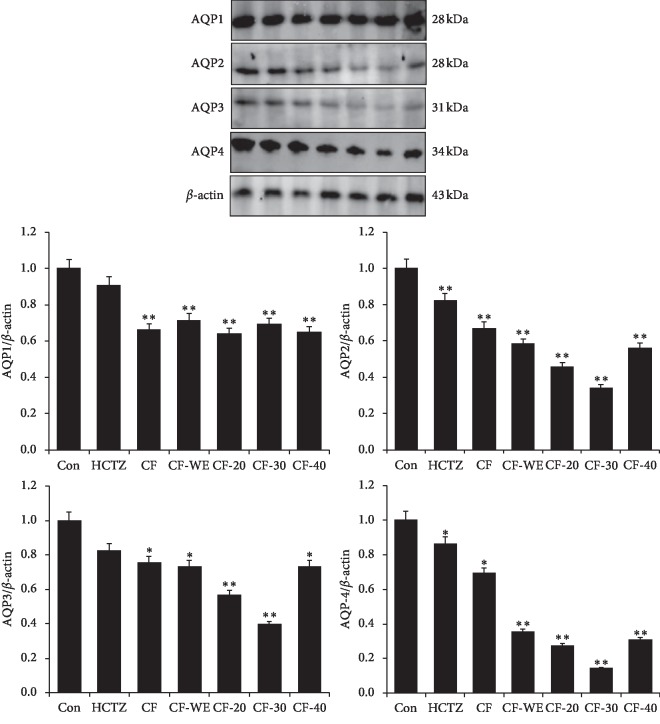
The expression of AQPs in the kidney as shown by Western blotting. Thirty minutes before drug administration, all mice were administered 0.2 mL/10 g body weight (BW) of isotonic saline to impose uniform water and salt load by oral gavage. Values are expressed as mean ± SD of three mice in each group. ^*∗*^*P* < 0.05 and ^*∗∗*^*P* < 0.01 compared to controls using Dunnett's *t*-test.

Aquaporins (AQPs) are membrane proteins that enable water molecules to pass through the lipid bilayer of cells. Four different aquaporins (i.e., AQP1, AQP2, AQP3, and AQP4) are expressed by the kidneys, and they play key roles in renal water reabsorption. Localization of AQP1, AQP2, AQP3, and AQP4 in the kidney of mice is shown in Figure 1. AQP1 is mainly distributed in the proximal convoluted tubule. AQP2 is abundant in the apical membrane of collecting duct. AQP3 and AQP4 are mainly distributed at the basolateral membrane of collecting duct. Western-blot images and immunofluorescence staining all showed that (Figure 2), compared with the control group, the groups receiving CF, CF-20, CF-30, and CF-40 all showed the decreased expression of AQP1, AQP2, AQP3, and AQP4 significantly (*P* < 0.05 and *P* < 0.01), with the strongest effects seen in response to CF-20, CF-30, and CF-40. In addition, hydrochlorothiazide also decreased the expression of AQP2 and AQP4 significantly (*P* < 0.05 and *P* < 0.01, respectively), and although it did not reduce the expression of AQP1 and AQP3 significantly, a trend toward reduction could be observed.

### 3.6. Effect on Apoptosis-Related Proteins in the Kidney

**Figure 3:**
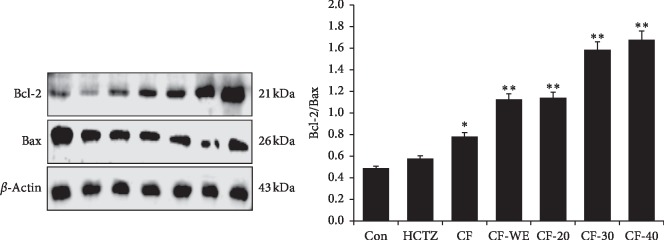
The expression of apoptosis-related proteins in the kidney tested by the Western-blot method. Thirty minutes before drug administration, all mice were administered 0.2 mL/10 g body weight (BW) of isotonic saline to impose uniform water and salt load by oral gavage. Values are expressed as mean ± SD of three mice in each group. ^*∗*^*P* < 0.05 and ^*∗∗*^*P* < 0.01 compared to controls using Dunnett's *t*-test.

In mammals, Bcl-2 and its related proteins play an important role in the regulation of apoptosis. The Bcl-2/Bax ratio determines the progress of apoptosis: When ratios are increased, apoptosis is inhibited, whereas decreased ratios stimulate apoptosis. The effect of the different fractions extracted from *C. flabellata* on apoptosis-related proteins is shown in Figure 3. Compared with the control group, those presented with CF, CF-WE, CF-20, CF-30, and CF-40 fractions all showed increased Bcl-2/Bax ratios potentially indicating inhibition of apoptosis in the kidney (*P* < 0.05 and *P* < 0.01).

### 3.7. Effect on MAPK Signaling Pathway-Related Proteins in the Kidney

**Figure 4:**
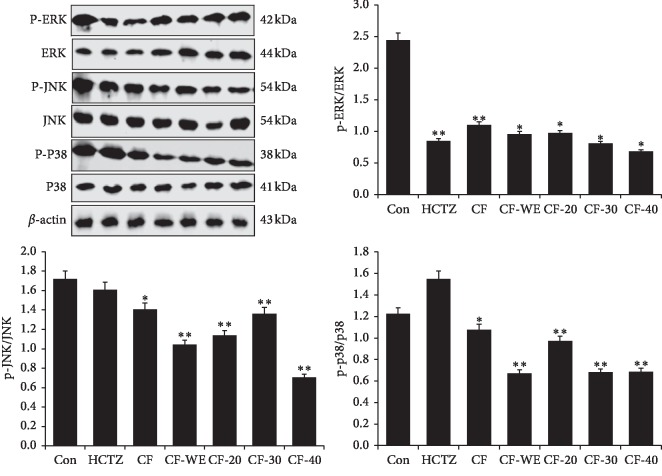
The expression of MAPK signaling pathway-related proteins in the kidney tested by the Western-blot method. Thirty minutes before drug administration, all mice were administered 0.2 mL/10 g body weight (BW) of isotonic saline to impose uniform water and salt load by oral gavage. Values are expressed as mean ± SD of three mice in each group. ^*∗*^*P* < 0.05 and ^*∗∗*^*P* < 0.01 compared to controls using Dunnett's *t*-test.

The MAPK signaling pathway is a signal transduction pathway that is present in various animal cell types. It includes three kinase complexes: p38 MAPK, c-Jun amino-terminal kinase (JNK), and extracellular signal-regulated kinase (ERK). We examined phosphorylation levels of ERK, JNK, and p38 in the kidney (Figure 4). Compared with the control group, the results for the groups receiving CF, CF-WE, CF-20, CF-30, and CF-40 showed significantly reduced ratios of p-ERK/ERK, p-JNK/JNK, and p-p38/p38 significantly (*P* < 0.05, *P* < 0.01), while the ratio of p-ERK/ERK was reduced the most. The effects of CF on ratios of p-JNK/JNK and p-p38/p38 were not as good as the other *C. flabellata* extracts. However, HCTZ also lowered the ratio of p-ERK/ERK and increased the ratio of p-p38/p38.

## 4. Discussion

As conceptualized in traditional Chinese medicine, *C. flabellata* is known to exhibit many effects, including stimulation of the blood circulation and detoxification, relief of swelling and pain, and reductions of heat and dampness [1]. It has been shown that the decoctions of this plant have anti-inflammatory, antibacterial, and antiviral activities by preliminary pharmacological tests [10]. Previous studies in our laboratory showed that *C. flabellata* may alleviate Alzheimer's disease and increase the urine volume in rat models. We conducted two studies to examine the effects of *C. flabellata* extracts on diuretic activity in mice systematically.

The acute diuretic experiment showed that medium and high doses of *C. flabellata* increased diuretic activity dose-dependently. To identify the active components of *C. flabellata*, i.e., with diuretic effects, we studied subchronic diuretic experiment with high doses of *C. flabellata* and its ethanol extracts over five days of administration in mice. The results showed that *C. flabellata* did have diuretic activity, and hydrochlorothiazide was used as a positive control drug with medium-strength diuretic activity. The results showed that water extracts, as well as 20%, 30%, and 40% ethanol extracts of *C. flabellata*, produced diuretic effects and that the latter extract (i.e., CF-40) produced robust long-term diuretic activity.

Urine osmolality reflects the kidneys' efficacy to concentrate and dilute urine and is often examined simultaneously with serum osmolality for clinical use. Our results showed that CF-40 and hydrochlorothiazide significantly increased the urine osmolality and concentration of Na^+^ and Cl^−^ in urine and decreased the concentration of Na^+^ in serum, but the serum osmolality did not reach statistical significance. The possible reason is CF-40 decreased the concentration of Na^+^ in serum while increased the urinary volume in mice. So, the osmolarity has no significant change. CF-20 and CF-30 only increased the Na^+^ concentration in urine. Although CF-WE did not increase urine osmolality and Na^+^ concentration significantly, a trend toward these effects could be observed. The lack of a statistically significant change in our study may be related to the modest number of animals that were tested. Hydrochlorothiazide is a classic diuretic, which acts primarily on the cortex of the ascending branches of the renal pulp as well as on the proximal end of the distal convoluted tubuli of the kidneys. It is known to increase the urinary excretion of sodium by inhibiting the Na^+^/Cl^−^ symporter (cotransporter system) [11]. In the present study, the results from osmolality and electrolyte levels in urine and serum showed that the diuretic mechanism of CF-40 is similar to that activated by hydrochlorothiazide.

Recent pharmacological studies indicate that the diuretic effect of drugs depends not only on the activity of ion channels that transport sodium and potassium but also on aquaporins that mediate water transport across cell membranes [12]. Aquaporins (AQPs) were identified as novel target molecule that might explain the efficacy of diuretic traditional Chinese medicines, which was confirmed by a number of quantitative studies on the diuretic effects of AQPs [13,14]. AQPs are intrinsic membrane proteins that are located in the cell membrane and control the ingress and egress of water between cells [15]. AQPs enable the body to quickly adjust the internal osmolality to maintain internal steady state. They are distributed throughout the body and are abundantly present in the kidney. One study reported that the renal expression levels of AQP1, AQP2, AQP3, and AQP4 are related to the reabsorption of water by the kidneys and the formation of urine [16]. *In vitro* experiments in mouse kidneys showed that the loss of AQP1 may reduce the reabsorption of water by the descending branch of the medullary canal, leading to polyuria in mice [17]. AQP2, AQP3, and AQP4 are mainly distributed in the renal collecting duct, and the expression levels of these aquaporins affect the concentration of urine, making AQPs relevant targets for many diuretics [18]. For example, AQP4 knockout mice did not show severe polyuria, but experiments proved them to be indispensable in transepithelial water transport mechanisms [19]. In the present study, compared with the control group, groups administered with CF, CF-20, CF-30, and CF-40 all significantly decreased their renal expression of AQP1, AQP2, AQP3, and AQP4 relative to a control group with saline treatment, an effect that was paralleled by a reduction of diuresis. This suggests that the diuretic effects of CF, CF-WE, CF-20, CF-30, and CF-40 are mediated by decreases in expression levels of AQPs 1–4.

Apoptosis plays an essential role in the maintenance of normal morphology and physiology of tissues and organs. The primary functions of the kidneys are to maintain the body's water, mineral, and pH balances and to protect the stability of the internal environment, thereby supporting the continuation of normal life activities. Intervention at the early stages of renal tissue apoptosis could potentially support and preserve renal functioning [20]. Apoptosis is relevant in the recovery of renal function in that inhibition of excessive apoptosis may help the preservation or recovery of organ function. In our study, daily oral administration of normal saline to mice imposed a burden on their kidneys. Accordingly, CF, CF-WE, CF-20, CF-30, and CF-40 all increased the Bcl-2/Bax ratio, which might inhibit the level of apoptosis in the kidneys. Accordingly, we hypothesize that the diuretic activity of CF, CF-WE, CF-20, CF-30, and CF-40 may be related to inhibiting apoptosis. It is interesting that *C. flabellata* extracts can inhibit the expression of apoptosis-related protein in the kidneys, while hydrochlorothiazide did not have this effect. The theory of traditional Chinese medicine records *C. flabellata* is home to the liver meridian, and the liver and the kidney have a common source. Therefore, *C. flabellata* may be beneficial to the kidneys to a certain extent. It is necessary for us to explore this issue further.

The MAPK cascade is one of the important cellular signaling systems that transduce extracellular stimuli to intracellular signals that reach the nucleus, initiating a series of cellular biological responses [21]. MAPK signal pathways contain four subfamilies: JNK/SAPK (c-Jun N-terminal or stress-activated protein kinases), ERK5/BMK (ERK/big MAP kinase 1), ERKs (extracellular signal-regulated kinases), and p38 MAPK. Studies have shown that the expression of AQP is closely related to the activation of the MAPK signaling pathway [22]. The expression of AQP3 can be regulated by the MAPK-ERK1/2 signal transduction pathway in human keratinocytes and embryonic cells [23]. AQP1 expression, JNKs, and p38-MAPKs are activated in astrocytes after MAPK-induced damage under various stress conditions (such as changes in osmotic pressure and metabolism) [24]. It also confirmed that, under hyperosmotic conditions, AQPs including AQP4 are regulated by MAPK [25]. Additionally, MAPK signaling pathways form important cellular signal transduction systems, with key roles in cell proliferation, growth, apoptosis, and intercellular synchronization [26]. Importantly, trichostatin A prevents TGF-*β*1-induced apoptosis in human renal tubular epithelial cells by inhibiting ERK activation [27]. Phosphorylation of JNK protein leads to inflammation and apoptosis in the renal tubules, which further impairs renal functioning [28]. Meanwhile, it has been determined that the activation of p38 MAPK leads to apoptosis of renal tubular cells, whereas inhibition of this pathway reduces tubular apoptosis and renal insufficiency [29,30]. In our study, CF-WE, CF-20, CF-30, and CF-40 all reduced phosphorylation levels of ERK, JNK, and p38, thereby inhibiting the activation of MAPK signaling pathways. Thus, CF-WE, CF-20, and CF-30 may inhibit the expression of AQPs and apoptosis-related proteins in the kidney by inhibiting the MAPK signaling pathway, which contributes to diuresis.

In the early stage, our laboratory analyzed the chemical constituents of the total extract and the 40% ethanol extract of *C. flabellata* by HPLC-MS. Results from mass spectrometry indicated the presence of six compounds in the total extract of *C. flabellata* including 3,4-dihydroxyphenylethanol-8-*O*-*β*-*D*-glucoside;4-hydroxyphenyl ethanol-8-*O*-*β*-*D*-glucoside; 3,4-dihydroxyphenylethanol-8-*O*-*β*-*D*-celosyl(1⟶3)-[*β*-*D*-glucosyl(1⟶6)]-4-*O*-caffeoyl-*β*-*D*-glucoside;5,3′,4′-trihydroxy-7,8-dimethoxy-6-C-[*β*-*D*-xylose-(1⟶2)]-*β*-*D*-glucoside flavonoid; 5,4′-dihydroxy-6,7-dimethoxy-8-C-[*β*-*D*-xylose-(1⟶2)]-*β*-*D*-glucosideflavonoid; and 5,3′,4′-trihydroxy-6,7-dimethoxy-8-C-[*β*-*D*-sucrose-(1⟶2)]-*β*-*D*-glucoside flavonoid. The first three compounds are phenylethanoid glycosides, the latter three are flavonoid glycosides, with a relatively high content. It was confirmed that phenylethanoid glycosides and flavonoid glycoside are the main components in *C*. *flabellate.* Also, six compounds of the 40% ethanol extract of *C. flabellata* could be identified, including 1′-*O*-*β*-*D*-(4-hydroxyphenethyl)-*β*-*D*-celyphate-(1⟶2)-glucoside; vanillic acid; 3,4-dihydroxyphenylethanol-8-*O*-*β*-*D*-glucoside; 1′-*O*-*β*-*D*-(3,4-dihydroxyphenylethyl)-*β*-*D*-celyose(1⟶3′)-*β*-*D*-glucose-(1⟶6′)-glucoside; and 5,4′-dihydroxy-6,7-dimethoxy-8-C-[*β*-*D*-xylose-(1⟶2)]-*β*-*D*-glucoside flavonoid.

The present study provided data for the use of different extracts from *C. flabellata* for diuretic applications in traditional Chinese medicine. The medium and high doses of the *C. flabellata* extract and CF-WE, CF-20, CF-30, and CF-40 were found to have a diuretic activity, with CF-40 having the longest lasting effect. Also, these *C. flabellata* extracts decreased the renal expression of AQPs, as well as the ratios of Bcl-2/Bax, p-ERK/ERK, p-JNK/JNK, and p-p38/p38.

## 5. Conclusion

Based on these results, we speculate that *C. flabellata* extracts may reduce the expression of AQP and apoptosis-related proteins in the kidney through inhibition of the MAPK signaling pathway, thereby also achieving diuretic effects.

## Figures and Tables

**Table 1:** Acute effect of oral administration of different *C. flabellata* extracts on urinary volume.

Group	Dose (mg/kg)	Urinary volume (mL/25 g)						
		1 h	3 h	5 h	7 h	9 h	11 h	Total in 11 h
Con	—	0.41 ± 0.06	0.34 ± 0.04	0.15 ± 0.05	0.12 ± 0.04	0.13 ± 0.10	0.13 ± 0.08	1.27 ± 0.12
HCTZ	40	0.57 ± 0.12^*∗∗*^	0.69 ± 0.09^*∗∗*^	0.31 ± 0.02^*∗∗*^	0.22 ± 0.04^*∗∗*^	0.14 ± 0.02	0.12 ± 0.04	2.06 ± 0.16^*∗∗*^
CF-L	77.5	0.35 ± 0.06	0.35 ± 0.06	0.20 ± 0.09	0.18 ± 0.11	0.22 ± 0.04	0.17 ± 0.08	1.46 ± 0.11
CF-M	155	0.37 ± 0.08	0.35 ± 0.05	0.28 ± 0.05^*∗*^	0.25 ± 0.04^*∗∗*^	0.25 ± 0.05^*∗*^	0.18 ± 0.05^*∗*^	1.67 ± 0.10^*∗*^
CF-H	310	0.44 ± 0.09	0.44 ± 0.04^*∗*^	0.30 ± 0.05^*∗∗*^	0.28 ± 0.10^*∗∗*^	0.27 ± 0.08^*∗*^	0.23 ± 0.11^*∗*^	1.96 ± 0.09^*∗*^

Values are expressed as mean ± SD of eight mice in each group. ^*∗*^*P* < 0.05 and ^*∗∗*^*P* < 0.01 compared to controls using Dunnett's *t*-test. Daily oral administration of CF, CF-WE, CF-20, CF-30, and CF-40 occurred on five consecutive days.

**Table 2:** Effects of different treatments on urine volume in mice 7 h after administration.

Group	Dose (mg/kg)	Urinary volume (mL/25 g)				
		First day	Second day	Third day	Fourth day	Fifth day
Con	—	1.09 ± 0.07	0.96 ± 0.18	1.04 ± 0.13	1.03 ± 0.11	1.02 ± 0.07
HCTZ	40	1.50 ± 0.21^*∗∗*^	1.56 ± 0.26^*∗∗*^	1.25 ± 0.12^*∗∗*^	1.27 ± 0.14^*∗∗*^	1.27 ± 0.19^*∗∗*^
CF	310	1.41 ± 0.09^*∗*^	1.32 ± 0.19^*∗*^	0.88 ± 0.18	1.08 ± 0.06	0.98 ± 0.23
CF-WE	82	1.42 ± 0.34^*∗*^	0.85 ± 0.27	0.96 ± 0.11	1.09 ± 0.15	0.98 ± 0.08
CF-20	20	1.45 ± 0.29^*∗*^	1.51 ± 0.20^*∗∗*^	0.96 ± 0.10	1.04 ± 0.09	0.97 ± 0.23
CF-30	20	1.54 ± 0.23^*∗∗*^	1.24 ± 0.16^*∗*^	0.89 ± 0.15	1.06 ± 0.09	0.89 ± 0.14
CF-40	36	1.56 ± 0.19^*∗∗*^	1.28 ± 0.18^*∗*^	1.22 ± 0.15^*∗*^	1.29 ± 0.11^*∗∗*^	1.27 ± 0.11^*∗∗*^

Values are expressed as mean ± SD of eight mice in each group. ^*∗*^*P* < 0.05 and ^*∗∗*^*P* < 0.01 compared to controls using Dunnett's *t*-test. Daily oral administration of CF, CF-WE, CF-20, CF-30, and CF-40 occurred on five consecutive days.

**Table 3:** Effects of *C. flabellata* extracts and hydrochlorothiazide on osmolality of urine and serum.

Group	Dose (mg/kg)	Urine osmotic (moms)	Plasma osmotic (moms)
Con	—	1025 ± 174.1	329.0 ± 23.6
HCTZ	40	1314.8 ± 180.1^*∗*^	328.7 ± 16.9
CF	310	1156.6 ± 156.1	331.6 ± 11.2
CF-WE	82	1212.0 ± 406.3	332.5 ± 12.7
CF-20	20	1233.8 ± 185.7	328.3 ± 7.0
CF-30	20	1209.0 ± 198.5	334.7 ± 12.1
CF-40	36	1275.6 ± 180.1^*∗*^	340.1 ± 8.9

Values are expressed as mean ± SD of eight mice in each group. ^*∗*^*P* < 0.05 and ^*∗∗*^*P* < 0.01 compared to controls using Dunnett's *t*-test. Daily oral administration of CF, CF-WE, CF-20, CF-30, and CF-40 occurred on five consecutive days.

**Table 4:** Effects of *C. flabellata* extracts and hydrochlorothiazide on the urine electrolyte concentration.

Group	Dose (mg/kg)	Urinary electrolyte concentration		
		Na^+^ (mmol/L)	K^+^ (mmol/L)	Cl^−^ (mmol/L)
Con	—	69.0 ± 6.5	76.4 ± 12.2	71.1 ± 4.6
HCTZ	40	86.6 ± 6.0^*∗*^	72.5 ± 15.0	84.5 ± 4.5^*∗∗*^
CF	310	66.9 ± 8.8	86.6 ± 1.8	73.3 ± 3.1
CF-WE	82	77.7 ± 6.9	77.9 ± 11.4	73.4 ± 4.4
CF-20	20	82.5 ± 11.9^*∗*^	65.4 ± 18.7	72.5 ± 7.2
CF-30	20	82.8 ± 5.6^*∗*^	81.3 ± 4.1	74.0 ± 3.3
CF-40	36	87.6 ± 9.7^*∗*^	76.5 ± 4.9	77.3 ± 4.3^*∗*^

Values are expressed as mean ± SD of eight mice in each group. ^*∗*^*P* < 0.05 and ^*∗∗*^*P* < 0.01 compared to controls using Dunnett's *t*-test. Daily oral administration of CF, CF-WE, CF-20, CF-30, and CF-40 occurred on five consecutive days.

**Table 5:** Effect of *C. flabellata* extracts and hydrochlorothiazide on the serum electrolyte concentration.

Group	Dose (mg/kg)	Serum electrolyte concentrations		
		Na^+^ (mmol/L)	K^+^ (mmol/L)	Cl^−^ (mmol/L)
Con	—	156.4 ± 8.1	8.7 ± 0.1	68.9 ± 1.1
HCTZ	40	143.4 ± 10.3^*∗*^	8.6 ± 0.1	68.0 ± 0.4
CF	310	157.5 ± 8.7	8.6 ± 0.3	68.4 ± 1.2
CF-WE	82	157.9 ± 5.9	8.5 ± 0.5	68.9 ± 0.5
CF-20	20	156.3 ± 9.0	8.0 ± 0.8	68.7 ± 0.5
CF-30	20	145.6 ± 10.2	8.2 ± 0.3	69.8 ± 0.7
CF-40	36	144.3 ± 18.4^*∗*^	8.4 ± 0.3	68.8 ± 0.6

Values are expressed as mean ± SD of eight mice in each group. ^*∗*^*P* < 0.05 and ^*∗∗*^*P* < 0.01 compared to controls using Dunnett's *t*-test. Daily oral administration of CF, CF-WE, CF-20, CF-30, and CF-40 occurred on five consecutive days.

## Data Availability

The data used to support the findings of this study are available from the corresponding author upon request.

## Supplementary Materials

Supplementary Materials The graphical abstract summarizes the research contents and main innovations of the article.
