# Supplementary material for: Corallodiscus flabellata B.L. Burtt Extracts Stimulate Diuretic Activity and Regulate the Renal Expression of Aquaporins
Source: Evid Based Complement Alternat Med. 2020 Feb 27;2020:6020817. doi: 10.1155/2020/6020817 (PMC7064869; doi:10.1155/2020/6020817)

**Graphical Abstract**

*Corallodiscus flabellata* B.L. Burtt extracts have a diuretic activity. They may reduce the expression of AQP and apoptosis-related proteins in the kidney through inhibition of MAPK signaling pathway, thereby achieving diuretic effects.


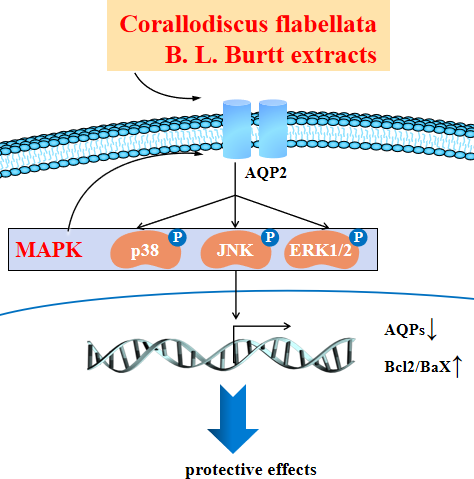

Supplement: Supplementary Materials — The graphical abstract summarizes the research contents and main innovations of the article. [file 6020817.f1.doc]
